# Draft Genome Sequence of the Globally Distributed Cockroach-Infecting Fungus Herpomyces periplanetae Strain D. Haelew. 1187d

**DOI:** 10.1128/MRA.01458-19

**Published:** 2020-02-06

**Authors:** Danny Haelewaters, Alicja Okrasińska, Michał Gorczak, Donald H. Pfister

**Affiliations:** a Farlow Reference Library and Herbarium of Cryptogamic Botany, Harvard University, Cambridge, Massachusetts, USA; b Department of Biology, Research Group Mycology, Ghent University, Ghent, Belgium; c Department of Molecular Phylogenetics and Evolution, Faculty of Biology, University of Warsaw, Warsaw, Poland; Vanderbilt University

## Abstract

Herpomyces periplanetae is an obligate biotroph of Periplaneta americana, the American cockroach. Its nearly cosmopolitan distribution is shaped by its globally invasive host and the international pet trade. Here, we report the draft genome sequence of *H. periplanetae*, based on a thallus from *P. americana* collected in Cambridge, Massachusetts.

## ANNOUNCEMENT

*Herpomyces* (Ascomycota, Pezizomycotina, Laboulbeniomycetes, Herpomycetales, Herpomycetaceae) is a genus of biotrophic fungi obligately associated with cockroaches (Arthropoda, Blattodea) (1–3). The distribution of at least some *Herpomyces* taxa has greatly expanded because of their globally invasive host species, which are used in the international pet trade (4). Recent molecular phylogenetic analyses have shown that *Herpomyces* species are distinct from members of the order Laboulbeniales, in agreement with developmental and morphological evidence (3, 5, 6). Species of both Herpomycetales and Laboulbeniales are unique among related fungi in that they do not form hyphae but instead produce multicellular units of hundreds to thousands of cells, referred to as “thalli,” through coordinated cell divisions from a single ascospore (3, 6–8). To date, these fungi have not been grown apart from their host; DNA needs to be isolated from individual thalli removed from the host (9–11).

Here, we sequenced and assembled the genome of H. periplanetae, isolate D. Haelew. 1187d, based on material removed from a Periplaneta americana specimen collected in Cambridge, Massachusetts (3). This genome is based on a single thallus (with three perithecia). Isolation and whole-genome amplification of genomic DNA (gDNA) were done using the REPLI-g single cell kit (Qiagen, Stanford, CA) (3). The single thallus was removed from the host and placed in a 0.2-ml PCR tube with 2.0 μl of phosphate-buffered saline (PBS) solution. After adding 1.5 μl of prepared denaturation (D2) solution, the tube was incubated at 65°C for 20 min, immediately followed by the addition of 1.5 μl of neutralizing REPLI-g STOP solution. Whole-genome amplification by multiple displacement amplification (MDA) was done in a 25-μl reaction mixture with 14.5 μl of REPLI-g reaction buffer, 1.0 μl of REPLI-g DNA polymerase, 4.5 μl of double-distilled water (ddH_2_O), and 5.0 μl of denatured DNA, under the following cycling conditions: incubation at 30°C for 6 h, followed by inactivation of the DNA polymerase at 65°C for 3 min. Following extraction and purification by ethanol precipitation, gDNA concentration and integrity were evaluated by agarose gel electrophoresis and using both a NanoDrop ND-1000 spectrophotometer (Thermo Fisher Scientiﬁc, Waltham, MA) and a Qubit double-stranded DNA (dsDNA) broad-range assay kit on a Qubit Fluorometer (Thermo Fisher Scientiﬁc). The NanoDrop values were within the required range for further processing (*A*_260_/*A*_280_ = 1.79, *A*_260_/*A*_230_ = 2.01), and the gDNA concentration as measured by the Qubit was 4.62 ng/μl. Identity and DNA isolation were validated by the amplification and sequencing of the following three ribosomal DNA fragments (3, 12): the small subunit (SSU) (GenBank accession number MG438331), internal transcribed spacer (ITS) (MG438309), and large subunit (LSU) (MG438359). Library preparation was done using the PrepX DNA library kit on the Apollo NGS library prep system (TaKaRa Bio, Mountain View, CA) following the manufacturer’s specifications and with 50 μl of gDNA as the starting volume. Paired-end sequencing (2 × 125 bp) was performed on an Illumina HiSeq 2500 instrument at the Harvard University Bauer Core Facility, resulting in a total of 9,793,875 raw paired-end reads.

Read quality was assessed using FastQC version 0.11.5 (13). Adapters were removed using Trimmomatic version 0.32 (14). Low-quality sequences were not trimmed, because this resulted in a poorer assembly (with more contigs and a lower *N*_50_ value). The remaining 8.5 million Illumina reads were *de novo* assembled using SPAdes version 3.10.1 (15) with progressive k-mer numbers, ranging from 21 to 117. The final genome assembly included 968 contigs (959 scaffolds), of which 632 (628) were longer than 1 kbp. After discarding scaffolds shorter than 1 kbp, the size of the genome was estimated to be 15,006,634 bp, with the length of the largest contig being 347,628 bp, an *N*_50_ value of 95,243 kbp, an *L*_50_ value of 50, and a GC content of 40.23%. These statistics were calculated using QUAST version 4.5 (16). To assess the completeness of our genome, we used BUSCO version 3.0.2 (17) with the fungal_odb9 database and a default species as the model. We retrieved 85% out of 290 orthologs present in the database (237 complete, 11 fragmented). This genome of *H. periplanetae* will enable studies of fungal genome evolution, host-parasite evolutionary genomics, patterns of speciation, and thallus development.

### Data availability.

The whole-genome shotgun sequencing project for the *Herpomyces periplanetae* isolate D. Haelew. 1187d has been deposited at DDBJ/ENA/GenBank under the accession number WMKJ00000000. The version described in this paper is version WMKJ01000000. The Illumina raw reads have been deposited at the Sequence Read Archive under accession number SRX7128323.
